# Unique Characteristics and Applications of Reverse Superior Labial Artery Island Flaps: A Case Series

**DOI:** 10.3390/medicina58081012

**Published:** 2022-07-28

**Authors:** Aleksander Zwierz, Krystyna Masna, Marcin Perczak, Paweł Burduk

**Affiliations:** 1 Department of Otolaryngology, Phoniatrics and Audiology, Faculty of Health Sciences, Ludwik Rydygier Collegium Medicum, Nicolaus Copernicus University, 85-168 Bydgoszcz, Poland; krymasna@gmail.com (K.M.); pburduk@wp.pl (P.B.); 2 Specialist Dental Practice Marcin Perczak, Nowa 15, 85-119 Bydgoszcz, Poland; perenko@wp.pl

**Keywords:** facial reconstruction, reverse superior labial artery flap, reverse flap, superior labial artery

## Abstract

*Background*: The reverse superior labial artery (rSLA) island flap can be used to reconstruct the cheek, ala, columella and vestibule of the nose when other techniques appear insufficient or impractical. The aim of this case series was to present applications of rSLA pedicle flaps in the post-ablative oncologic reconstruction of the face. *Patients and Methods*: Using a retrospective case-series study design, the investigators enrolled a cohort of patients undergoing procedures involving rSLA flaps treated at a Polish Otolaryngology Department for facial reconstruction after tumour excision. The main outcomes were functional and aesthetic aspects. Descriptive statistics were computed as appropriate. *Results and Conclusions*: The use of rSLA flaps allows surgeons to obtain a large skin island with only minimal cosmetic and functional alterations. In all of the cases in this series, the use of this pedicle flap resulted in both optimal healing and satisfactory cosmetic and functional outcomes.

## 1. Introduction

The concept of arterial reverse-flow island flaps was introduced in 1976 by Bostwick et al., who proposed the use of reverse-flow superficial temporal artery island skin flaps from the preauricular area for lateral forehead defect reconstruction [1]. Since then, many other concepts involving reverse-flow flaps have been proposed, mostly for extremity reconstruction [2,3,4,5,6,7,8].

**Figure 1:**
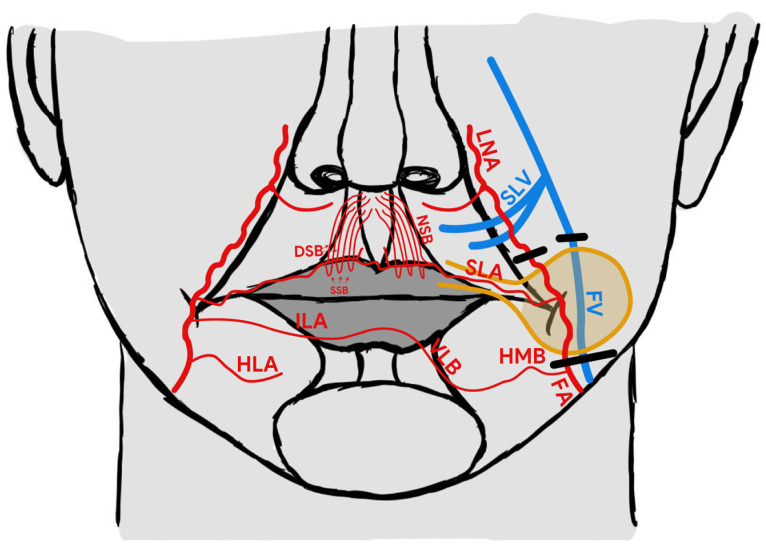
rSLA island flap anatomy. FA—facial artery, FV—facial vein, SLA—superior labial artery, SLV—superior labial vein, ILA—inferior labial artery, LNA—lateral nasal artery, HLA—horizontal labiomental artery, HMB—horizontal mental branch, VLB—vertical labiomental branch, NSB—nasal septal branches, DSB—deep septal branch, SSB—superficial septal branch. The black line indicates the location of the vessels’ ligation.

**Figure 2:**
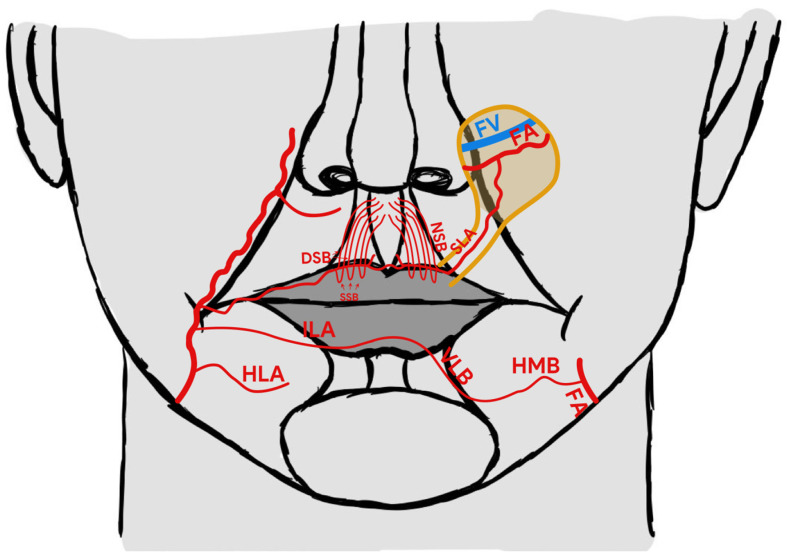
rSLA pedicle skin flap rotation into the donor site. FA—facial artery, FV—facial vein, SLA—superior labial artery, SLV—superior labial vein, ILA—inferior labial artery, HLA—horizontal labiomental artery, HMB—horizontal mental branch, VLB—Vertical labiomental branch, NSB—nasal septal branches, DSB—deep septal branch, SSB—superficial septal branch.

In 2015, Turan et al. suggested the reverse superior labial artery (rSLA) island flap as an alternative reconstructive method for use in relation to nose and medial cheek defects [9]. The concept of a reverse arterial blood flow to such flaps is based on current knowledge concerning the particular vascular anatomy [10,11]. More specifically, it is based on communication of both sides’ superior labial arteries and various types of communication between the inferior labial artery (ILA) or horizontal labiomental artery (HLA) on one side with the arteries on the other side. Many studies have described a variable arterial pattern of the lower lip [11,12,13]. The two most frequent types of blood supply described by Lee at al. are presented on Figure 1. The anastomosis between the right and left side formed by side-crossing artery branches like the horizonal mandibular branch and vertical labial branch of the horizontal labiomental artery may be also important for reverse feeding of the pedicle flap (Figure 1). In the case of ligation of the facial artery on the one side, it provides an influx of blood from the other facial artery supplying the angiosome of the contralateral SLA and, thanks to the described communication, may supply blood from the ligated facial artery (Figure 1 and Figure 2) [12,13].

Apart from different directions of blood flow, this flap composed of a skin island in the nasolabial fold area may prove useful when other reconstructive techniques are insufficient or impractical as a result of tumour localisation on the course of the planned pedicle vessels or when previously performed surgery may have destroyed the pedicle or it is not possible to rotate the pedicle of other flaps (Figure 2).

In this case series, we present three applications of rSLA island flaps for the reconstruction of facial skin defects following oncological resections.

## 2. Case Report

We present three cases of patients who underwent reconstruction of the medial cheek, nasal ala and/or vestibule of the nose following oncological resection.

### 2.1. Case 1

**Figure 3:**
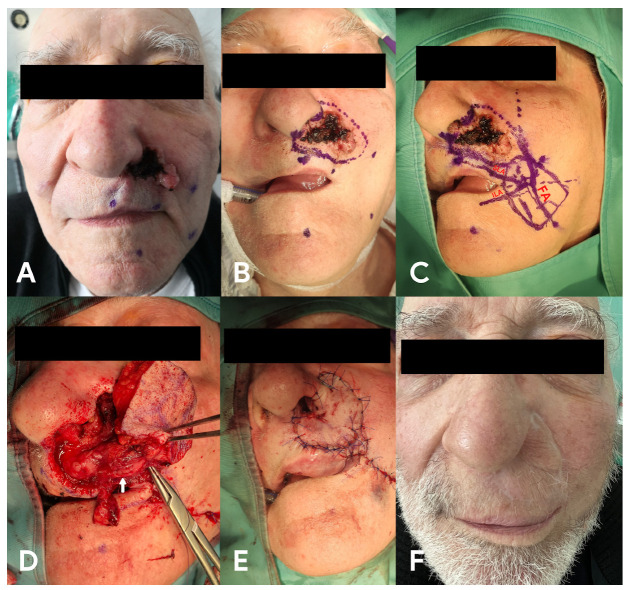
Basal cell carcinoma of the left ala of the nose and left cheek. (**A**)—a view of the tumour, (**B**)—range of the resection, (**C**)—rSLA flap planning, FA—facial artery, SLA—superior labial artery, ILA—inferior labial artery, (**D**)—pedicle of the flap (white arrow), (**E**) final reconstruction, and (**F**) patient 12 months after the surgery.

A 76-year-old male was admitted to our clinic with a history of eroding malignant lesions due to basal cell carcinoma (BCC). The tumour involved the left ala of the nose and left cheek, and its dimensions were approximately 25 × 23 mm (Figure 3A). The day before surgery Doppler was used and the facial artery and its branches were marked. In the presented case, we found the labiomental artery on the left side to be quite large in horizontal diameter. We took photos of the patient’s face and printed them. We redrew the vessels during the flap planning. This schema was also adopted for the other presented cases. Since the Doppler ultrasonography showed good blood flow in both SLA and HLA, and we were concerned that the facial artery could be affected by the tumour in its further course, we planned reconstruction using the rSLA pedicle flap. Under general anaesthesia, total resection of the tumour with a 5–10 mm deep margin of macroscopically healthy tissue was performed. Moreover, the ala of the nose was reconstructed, and the defect was covered with an rSLA flap (Figure 3B–E). At the end of surgery and in the next postoperative days, we stung the flap and observed whether there was bleeding. The postoperative pathological examination confirmed the presence of BCC and, further, that all of the margins of the tumour were free from cancer infiltration. The patient was discharged from the clinic in good condition. A follow-up examination was conducted after 12 months (Figure 3F).

### 2.2. Case 2

**Figure 4:**
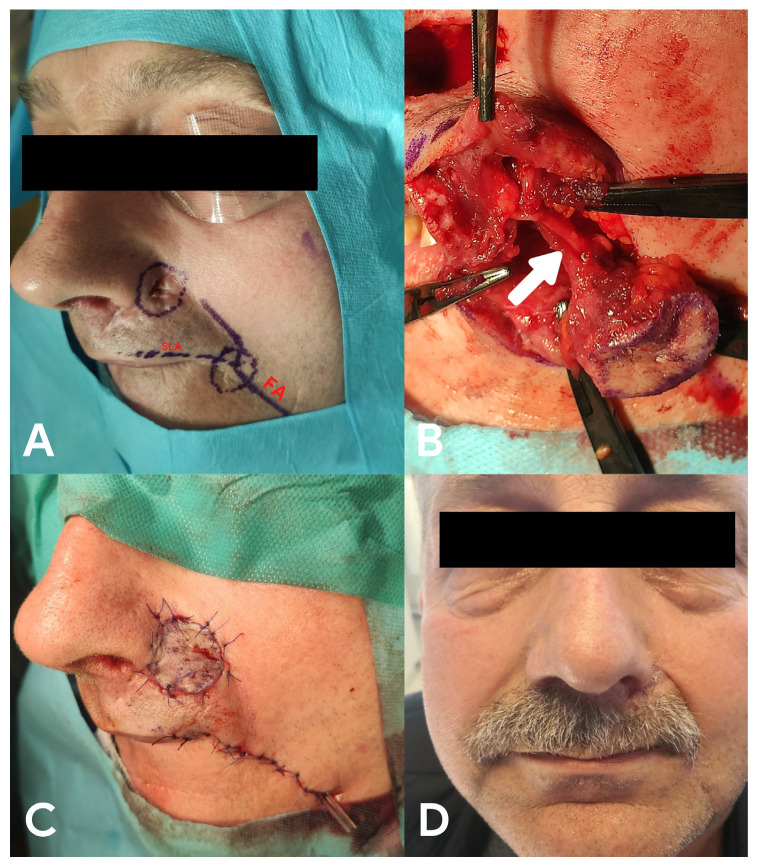
Recurrence of basal cell carcinoma of the upper part of the left nasolabial fold. (**A**) flap planning, FA—facial artery, SLA—superior labial artery, (**B**) pedicle of the flap (white arrow), (**C**) final reconstruction, and (**D**) patient 12 months after the surgery.

A 58-year-old male presented with ulcerative tumour recurrence after resection of BCC at the left nasal alar base and the upper part of the nasolabial fold 5 years previously (Figure 4A). Performed surgery might have destroyed the ateral nasal artery and alar branches of the facial artery and would hamper the formation of a vascular pedicle and creation of a V-Y fasciocutaneous flap. In this case, we used the rSLA flap due to the different origins of the pedicle vessels, which seemed to not be destroyed. The patient underwent tumour resection with a 5 mm free margin and subsequent reconstruction using an rSLA flap (Figure 4B,C). The flap completely healed, and his cosmetic and functional outcomes were good (Figure 4D).

### 2.3. Case 3

**Figure 5:**
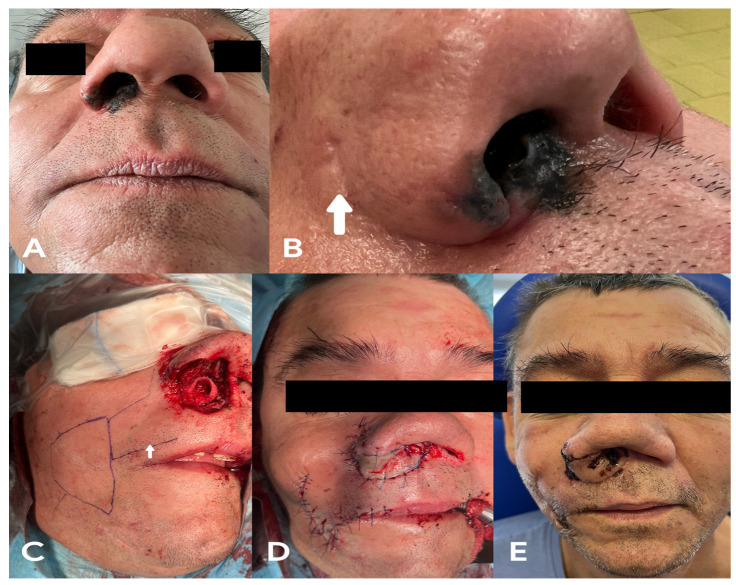
(**A**) melanoma of the right vestibulum and nasal cavity, (**B**) scar left by previous surgery (white arrow), (**C**) rSLA flap planning, (**D**) final reconstruction, and (**E**) patient one month after the surgery.

A 47-year-old male was admitted to the clinic due to a tumour of the right vestibulum and cavum of the nose (Figure 5A), which occurred three years after he had undergone fibroma-related resection of the right nasal cavity. His medical history was unavailable because his previous surgery had been performed in a different ear, nose and throat (ENT) clinic, although a scar was visible due to an incision in his right nasal alar groove (Figure 5B). Right-side lateral rhinotomy was performed, and the tumour was resected with a 5 mm free margin. The resection included the right ala of the nose; the fundus of the nasal vestibule and nasal cavity; the lateral nasal wall, including the right inferior nasal concha; the septum of the nose, including the right mucoid tissue and septal cartilage; and the nasal columella skin. The possibility of using different flaps was limited because of the previously performed surgery; lateral rhinotomy might destroy the lateral nasal artery, facial artery and its alar branches. Moreover, the reconstruction of the vestibule of the nose and nasal septum region following resection could again be problematic owing to difficulties associated with the pedicle rotation of the flap in the resected area. Therefore, in this case, the tissue defect was reconstructed using a huge rSLA skin island flap (Figure 5C,D). The flap completely healed and the patient’s cosmetic outcome was satisfactory (Figure 5E). The postoperative pathological examination indicated a diagnosis of melanoma. Thus, the patient underwent postoperative chemotherapy.

## 3. Discussion

The superior labial artery island flap technique originally proposed by Turan et al. allows surgeons to obtain a huge buccal skin island and, therefore, to perform the primary closure of the donor site with only minimal facial asymmetry [9]. Tanaka and Tajima stated that the surviving area of the reverse-flow flap is smaller than that of flaps with antegrade arterial flow, while the most important factor for island flap survival is adequate concomitant vena outflow [14]. This is puzzling because most anatomy textbooks agree that the SLA has no concomitant vein, as the superior labial vein (SLV) runs in a different direction—horizontally and laterally in the direction of the ala of the nose [12]. The SLV crosses the facial artery at a near right angle. Moreover, the SLV runs on the cutaneous side of the orbicularis oris muscle, whereas the SLA runs on the oral mucosal side [15]. Yet, Camuzard et al. found small venae concomitant to the facial artery [16]. In addition, Jing stated that in contrast to antegrade island flaps, venous drainage in the case of retrograde island flaps principally occurs through the inner and outer membrane of the artery and wall of the vein [17]. Therefore, it is extremely important to raise a thick tissue pedicle with a diameter of at least 6 mm that can support the blood outflow from the flap alongside the facial artery. We can confirm the findings reported by Turan et al. concerning more than 50 cases involving the rSLA technique, as we did not observe any partial or total flap loss, which indicates the ability an rSLA flap to create a huge skin island with good venosus blood outflow [9,18].

The reconstruction of tissue defects localised in the alar or vestibulum nasal region following resection is difficult because radical oncological surgery destroys important vascular structures such as the facial artery and its alar branch. This impedes the use of certain reconstructive techniques, for example, V-Y fasciocutaneous free-style perforator flaps of the facial angiosome. This was the situation presented in Case 1. Moreover, the previously performed surgery described in Cases 2 and 3 may have destroyed these vessels or created scar tissue that could hamper the formation of a vascular pedicle. Furthermore, cosmetic and functional reconstruction of the vestibule of the nose and nasal septum region following resection, which was presented in Case 3, could again be problematic owing to difficulties associated with the pedicle rotation of the flap in the resected area.

Despite the need to divide the risorius, zygomaticus major and orbicularis oris muscles to create the pedicle, we did not observe significant local morbidity after raising the flap or any functional dysfunction of the face. It should also be noted that the buccal branches of the facial nerve lie deeper than the facial artery, meaning that careful preparation of the flap should ensure that they are not destroyed [19].

The healing time is especially important in relation to oncological reconstructions. The work of di Summa et al. compared the outcomes of elbow reconstructions using reverse-flow and perforator-based flaps. In all of the cases, the effective coverage of the loss of the substance at the elbow was achieved. However, they concluded that the reverse-flow flap technique requires less operative time and shows better reliability, thereby resulting in significantly decreased hospitalisation time [5].

## 4. Conclusions

The findings of this case series indicate that the rSLA island flap technique may be a good option for the reconstruction of defects in the cheek, ala, vestibulum and septum nasi region following ablative surgery. It may be useful in the case of previously performed surgery, when other reconstructive options are not available, the performed resection destroyed other pedicle vessels or if the defect is localized in the vestibulum and septum nasi region. It allows the surgeon to obtain a large skin island and, therefore, to ensure minimal cosmetic and functional dererioration. The application of the technique proposed by Turan et al. and the creation of a pedicle that is a minimum of 6 mm in diameter should allow the flap to survive.

## Data Availability

Not applicable.
